# Supplementary material for: Skp2-mediated MLKL degradation confers cisplatin-resistant in non-small cell lung cancer cells
Source: Commun Biol. 2023 Aug 2;6:805. doi: 10.1038/s42003-023-05166-6 (PMC10397346; doi:10.1038/s42003-023-05166-6)
Supplement: Supplementary file 2 — Supplementary Information [file 42003_2023_5166_MOESM2_ESM.pdf]

Skp2-mediated MLKL degradation confers cisplatin-resistant in non-small cell lung cancer cells

Huiling Zhou[1,2*], Li Zhou[3*], Qing Guan[1,2], Xuyang Hou[1,2], Cong Wang[2], Lijun Liu[1,2], Jian Wang[1,2], Xinfang Yu[4], Wei Li[5], and Haidan Liu[1,2]

[1] Department of Cardiovascular Surgery, The Second Xiangya Hospital of Central South University, Changsha, Hunan, China [2] Clinical Center for Gene Diagnosis and Therapy, The Second Xiangya Hospital of Central South University, Changsha, Hunan, China [3] Department of Pathology, National Clinical Research Center for Geriatric Disorders, The Xiangya Hospital of Central South University, Changsha, Hunan, China [4] Department of Medicine, Baylor College of Medicine, Houston, TX 77030, USA [5] Department of Radiology, The Third Xiangya Hospital of Central South University, Changsha, Hunan, China

* These authors contributed equally

Correspondence to: Haidan Liu, haidanliu@csu.edu.cn

Wei Li, Weililx@csu.edu.cn

Supplementary Table 1
Supplementary Table 2
Supplementary figures:
Supplementary Figure 1
Supplementary Figure 2
Supplementary Figure 3
Supplementary Figure 4

Supplementary Table 1. Protein expression of Skp2 in NSCLC tissues and adjacent non-tumor tissues.

Tissue sample	No. of patients	Skp2		*p*- value
		Low	High	
Tumor	39	6	33	<0.0001*
Adjacent	39	28	11	

Chi-square test.

*p<0.05 indicates a significant association among the variables.

Supplementary Table 2. Protein expression of MLKL in NSCLC tissues and adjacent non-tumor tissues.

Tissue sample	No. of patients	MLKL		*p*- value
		Low	High	
Tumor	39	26	13	0.0002*
Adjacent	39	9	30	

Chi-square test.

*p<0.05 indicates a significant association among the variables.

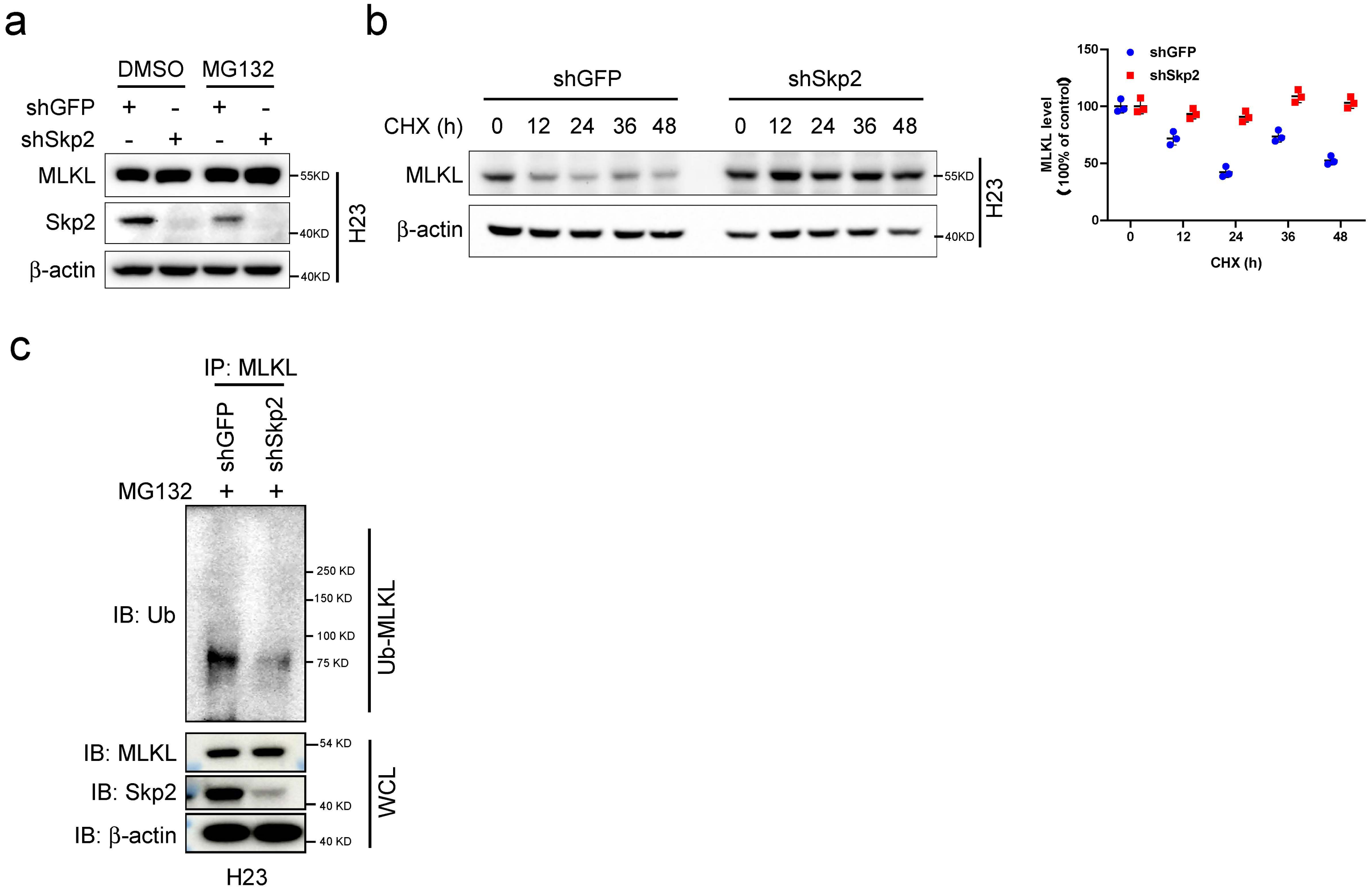

Supplementary Figure 1 Skp2 regulates the stability and ubiquitination of MLKL protein. a MLKL expression was analyzed in H23 cells expressing shGFP or shSkp2 and treated with MG132 (20 μM) for 12 h. WCEs were subjected to Western blot analysis. b H23 cells expressing shGFP or shSkp2 were subjected to cycloheximide chase (25 μg/mL for indicated times). WCEs were subjected to Western blot analysis. c Stable Skp2 knockdown H23 cells were treated with MG132 (20 mM) for 6 h, WCEs were prepared and subjected to MLKL ubiquitination analysis using an ubiquitin antibody.

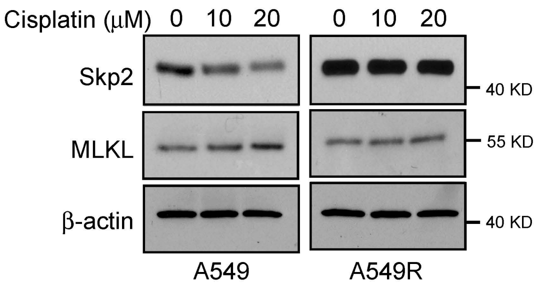

Supplementary Figure 2 Effect of acute exposure to cisplatin on Skp2 and MLKL protein levels in A549 and A549R cells. Cells were treated with indicated doses of cisplatin for 24 h, WCEs were subjected to Western blot analysis.

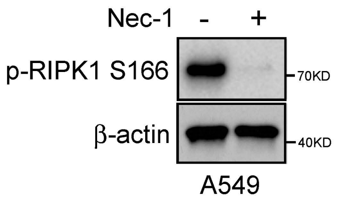

Supplementary Figure 3 Effect of necrostatin-1 (Nec-1) on the phosphorylation of RIPK1 at Ser166 in A549 cells. Cells were treated with DMSO or Nec-1 (50 μM) for 24 h, WCEs were subjected to Western blot analysis.

Fig. 1a

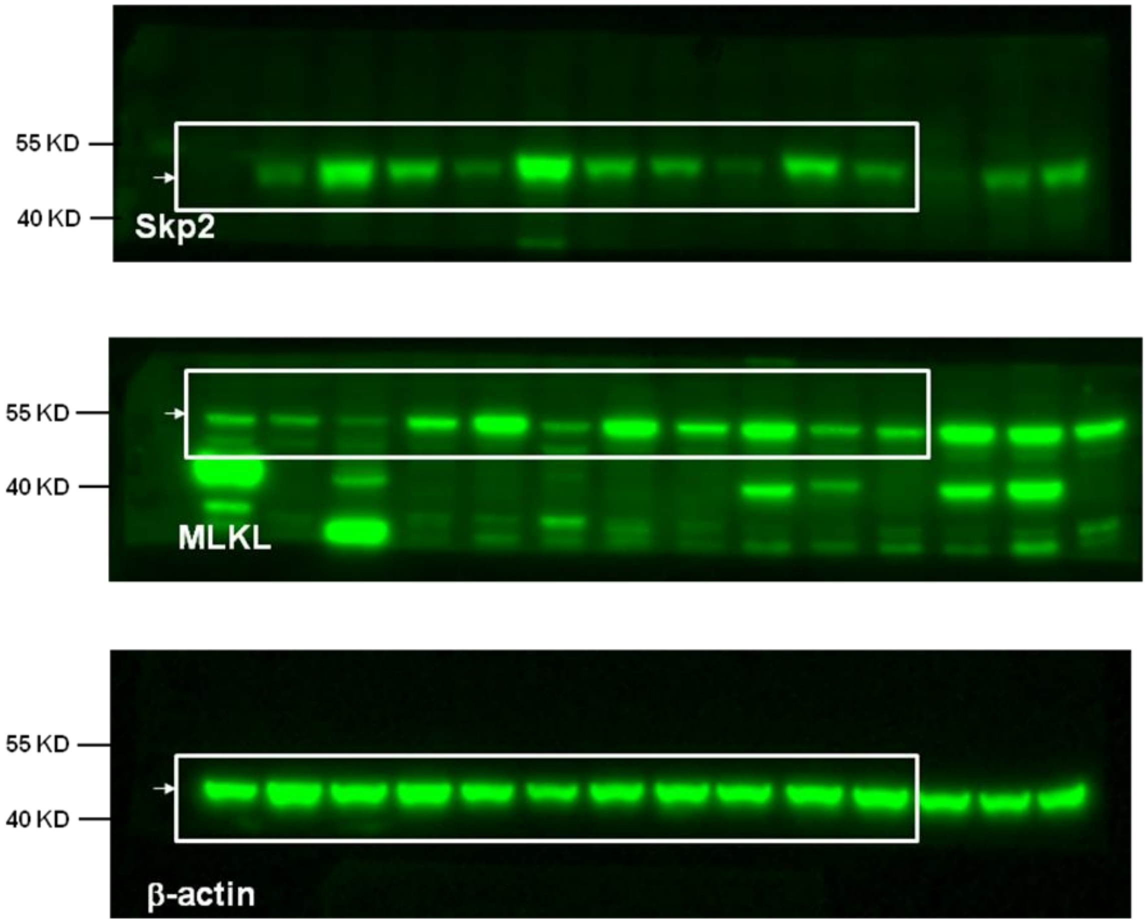

By FluorChem FC2 imaging system (Alpha innotech, USA)

Fig. 1c

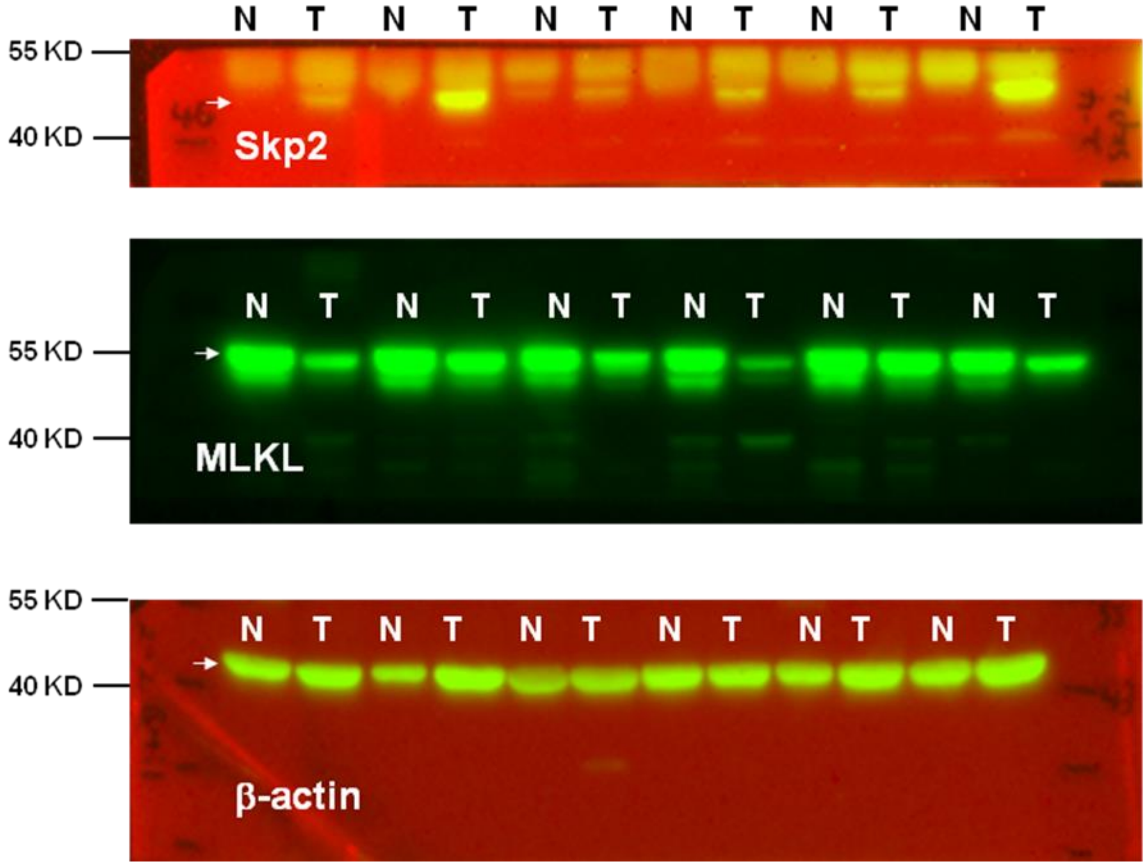

By FluorChem FC2 imaging system (Alpha innotech, USA)

Fig. 1e

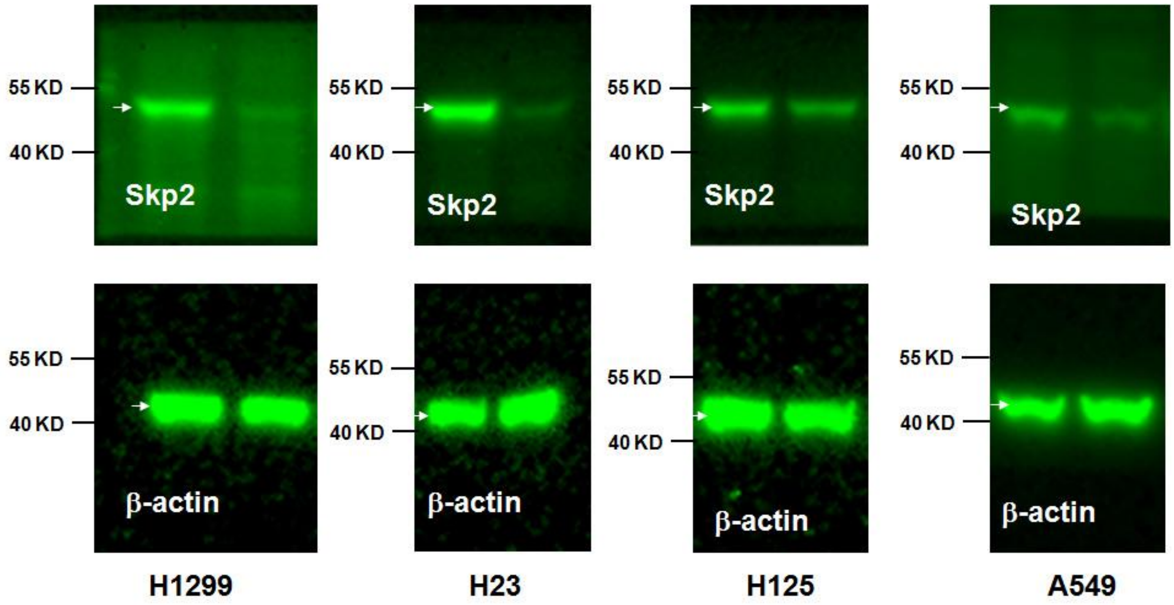

By FluorChem FC2 imaging system (Alpha innotech, USA)

Fig. 3a

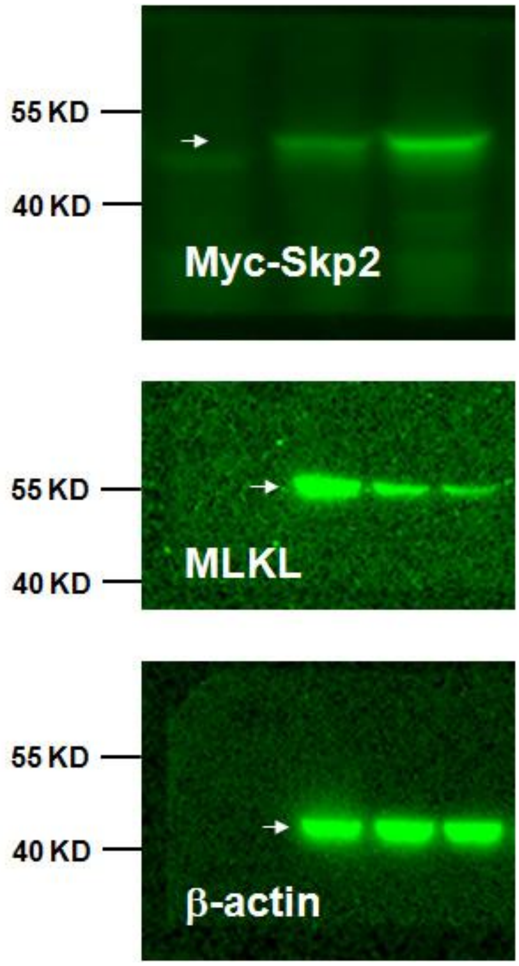

By FluorChem FC2 imaging system (Alpha innotech, USA)

Fig. 3b

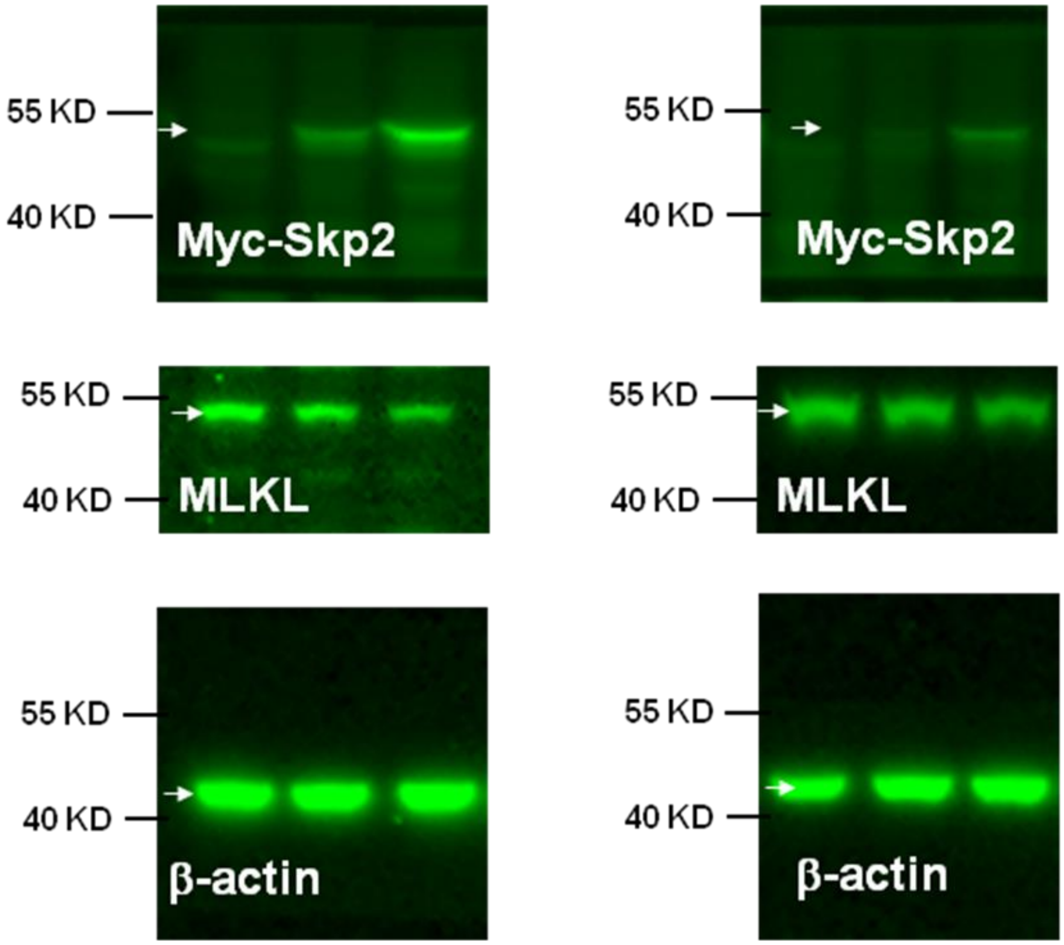

By FluorChem FC2 imaging system (Alpha innotech, USA)

Fig. 3c

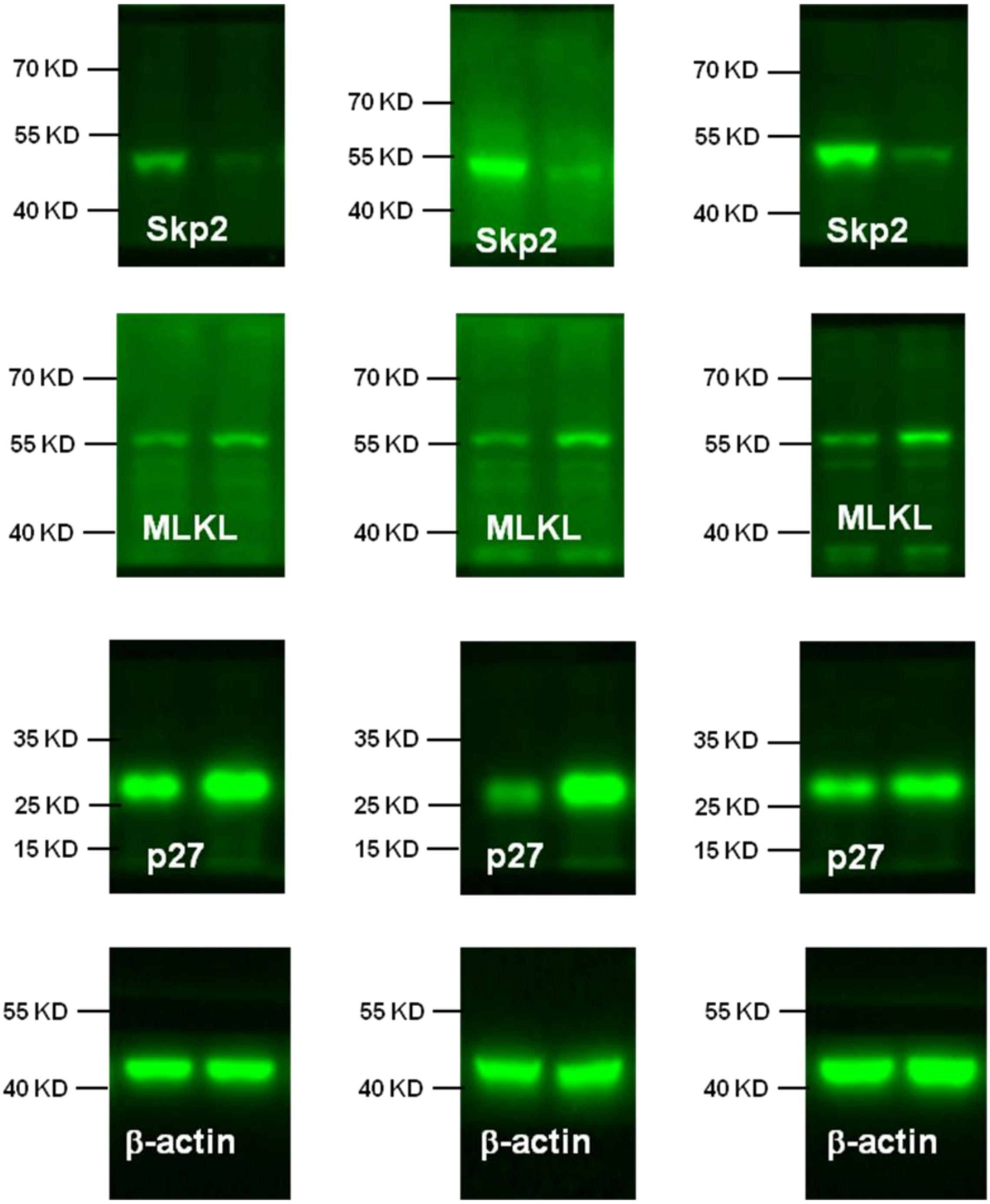

By FluorChem FC2 imaging system (Alpha innotech, USA)

Fig. 3d

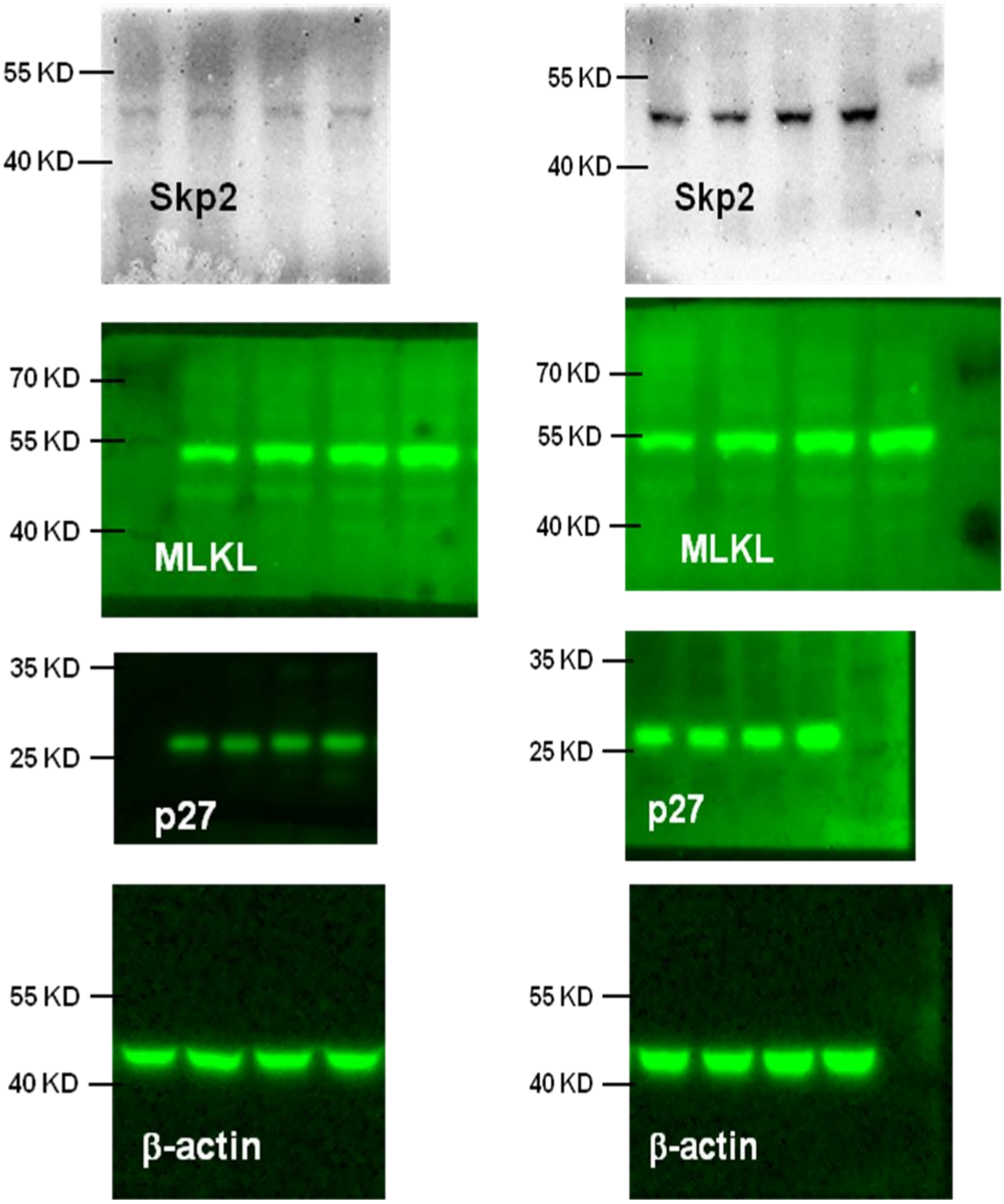

By FluorChem FC2 imaging system (Alpha innotech, USA)

Fig. 4a

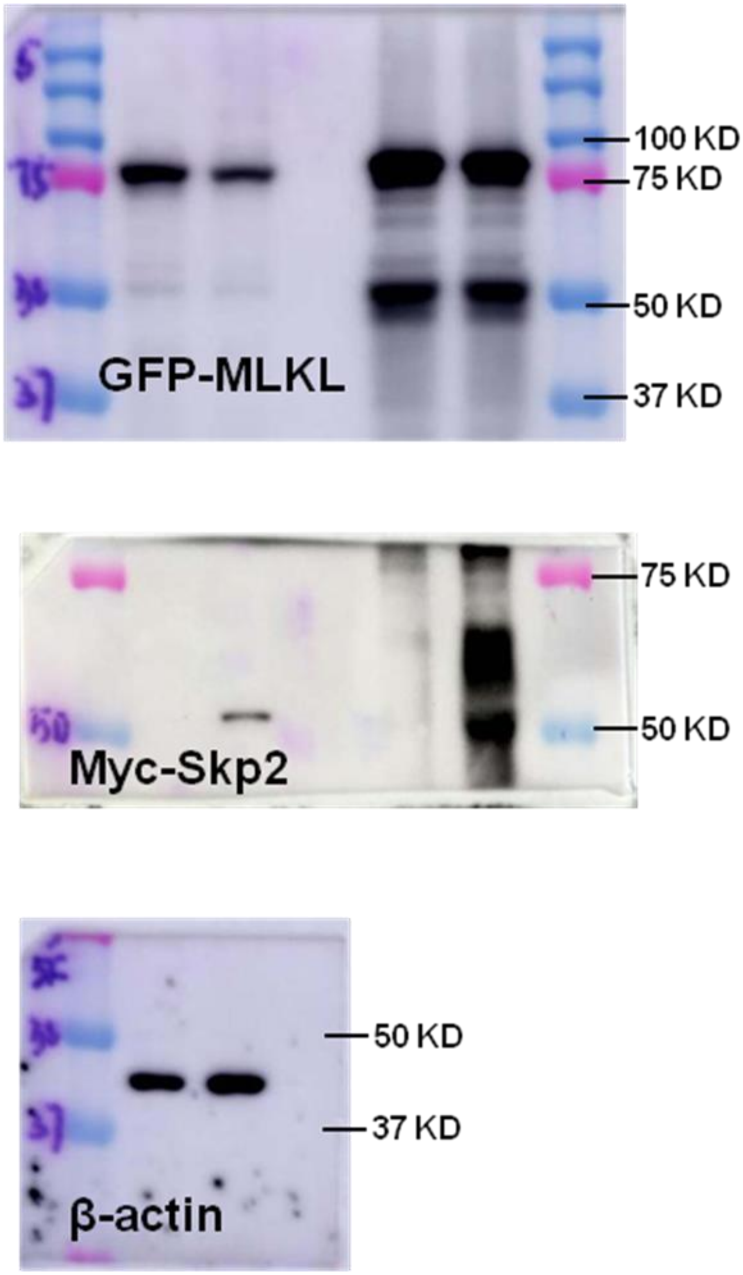

By AMERSHAM imageQuant800 system (GE, USA)

Fig. 4b

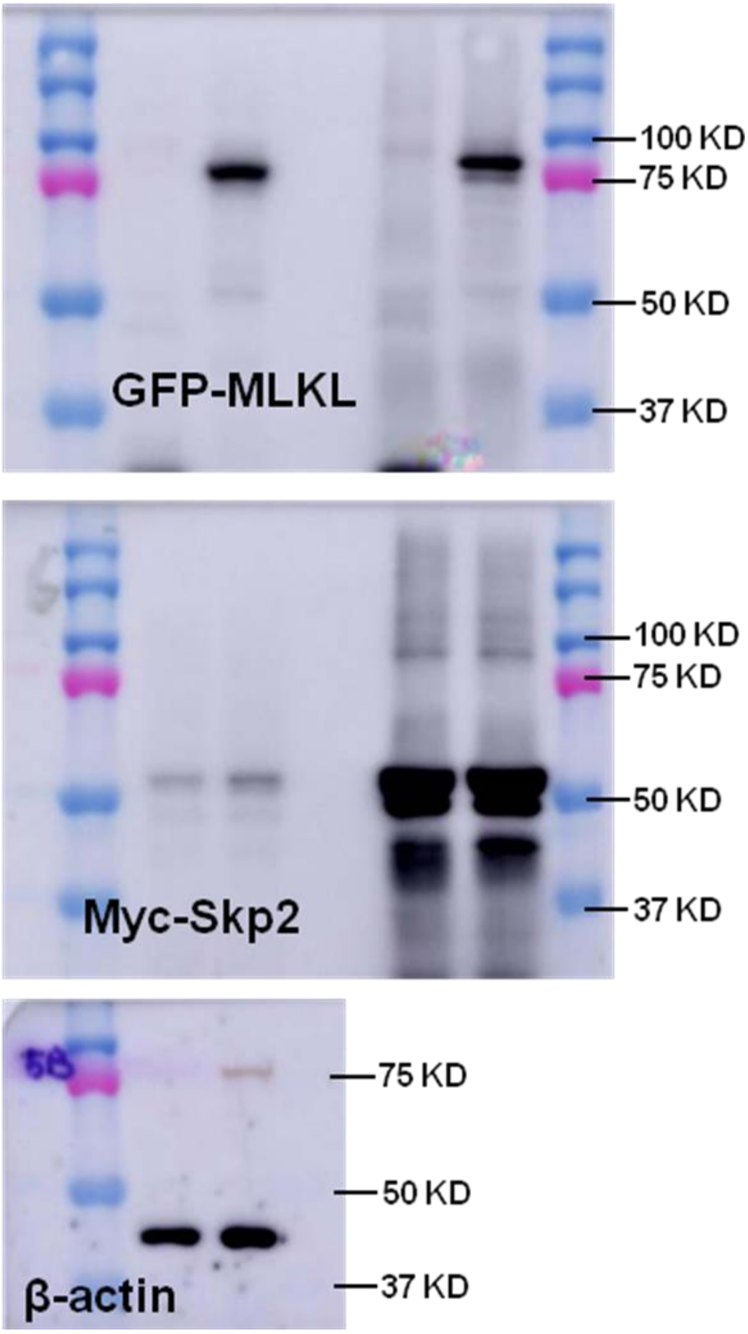

By AMERSHAM imageQuant800 system (GE, USA)

Fig. 4c

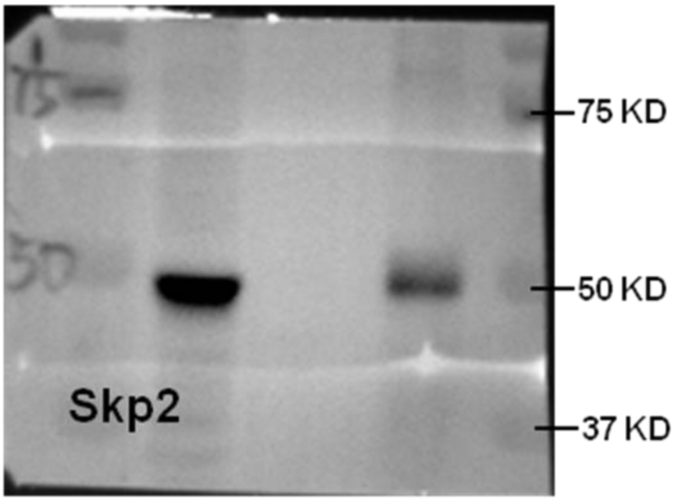

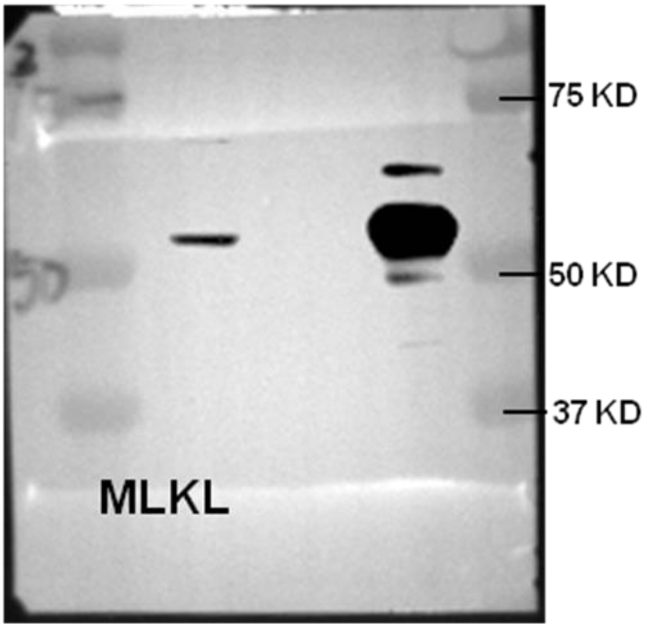

By AMERSHAM imageQuant800 system (GE, USA)

Fig. 4d

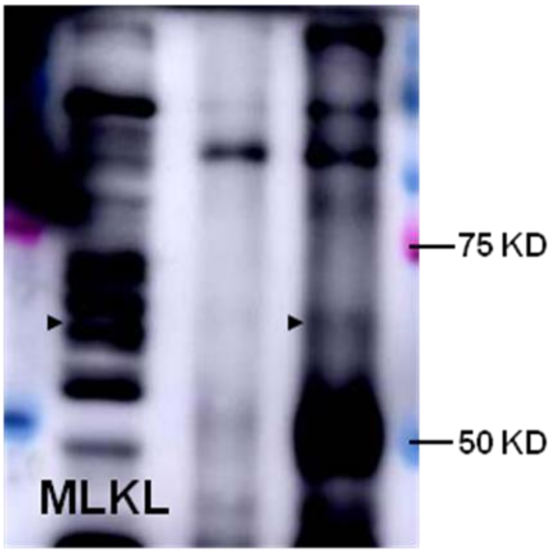

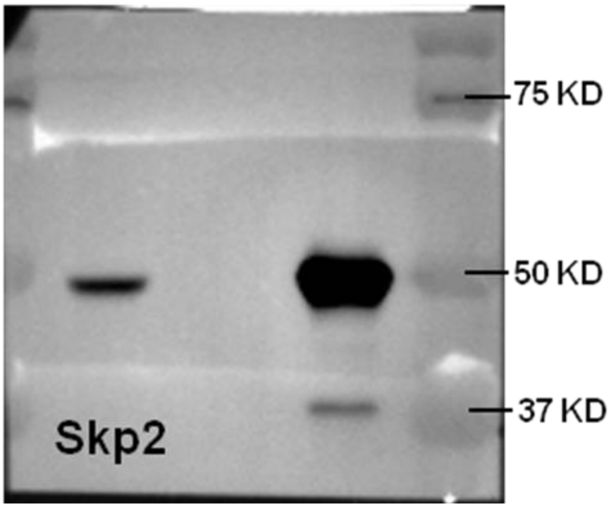

By AMERSHAM imageQuant800 system (GE, USA)

Fig. 4e

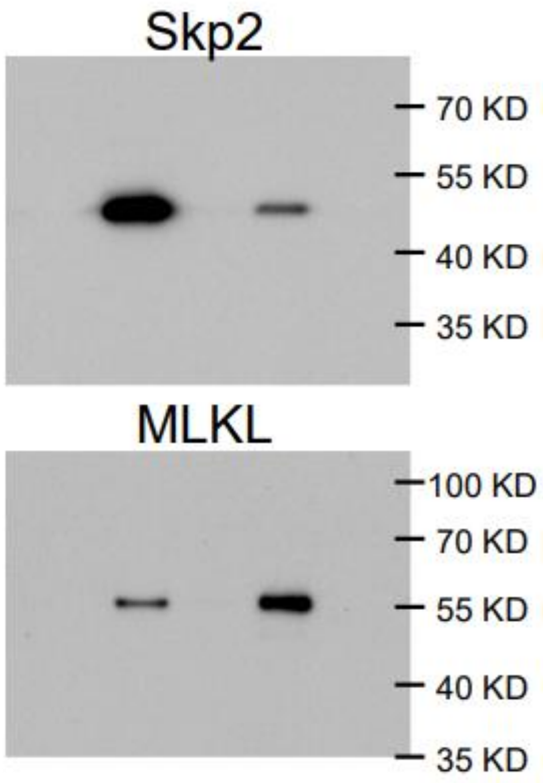
Skp2
70 KD
55 KD
40 KD
35 KD
MLKL
100 KD
70 KD
55 KD
40 KD
35 KD

Fig. 4f

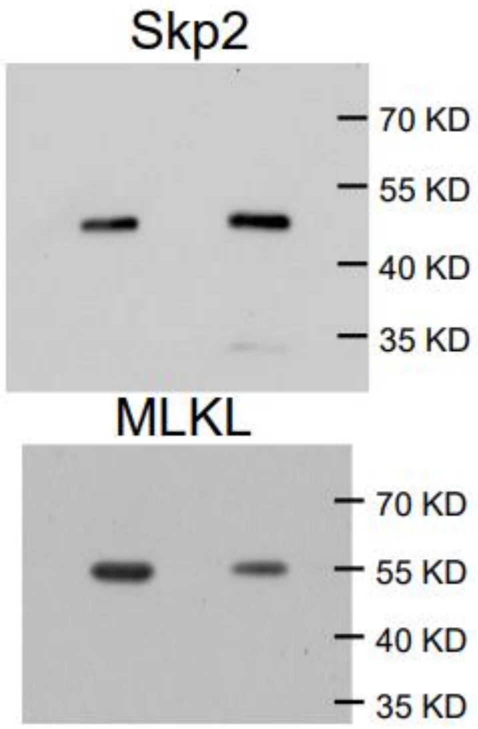
Skp2
70 KD
55 KD
40 KD
35 KD
MLKL
70 KD
55 KD
40 KD
35 KD

Fig. 4g

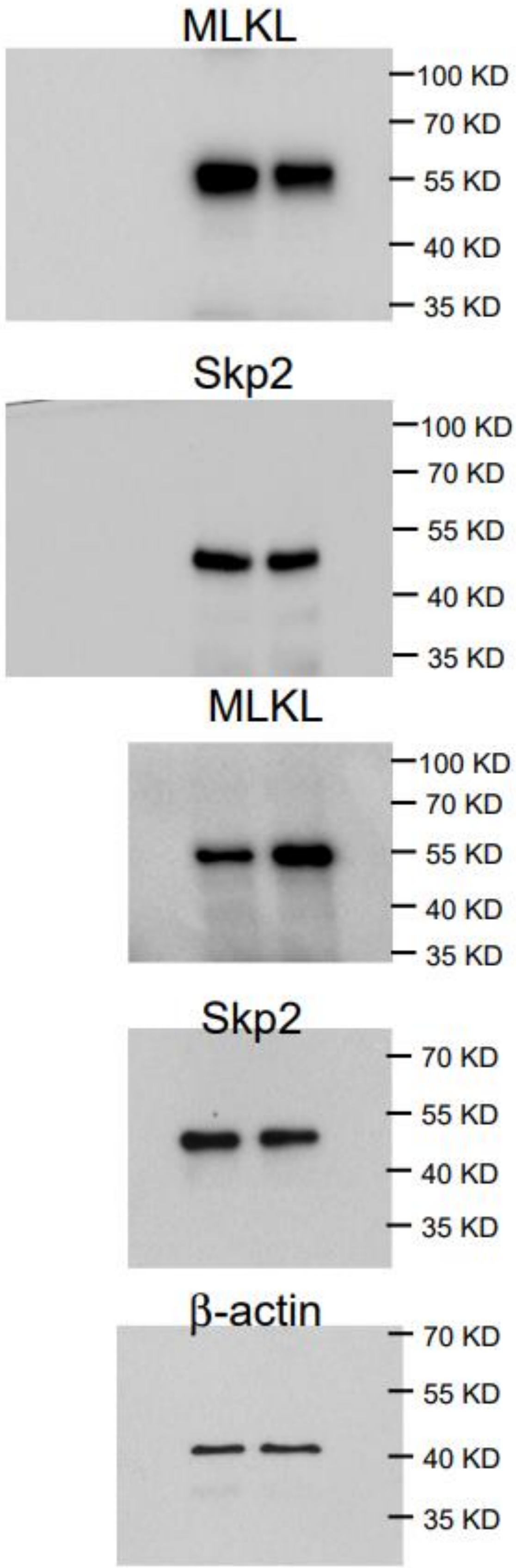

MLKL
100 KD
70 KD
55 KD
40 KD
35 KD
Skp2
100 KD
70 KD
55 KD
40 KD
35 KD
MLKL
100 KD
70 KD
55 KD
40 KD
35 KD
Skp2
70 KD
55 KD
40 KD
35 KD
β-actin
70 KD
55 KD
40 KD
35 KD

Fig. 5a

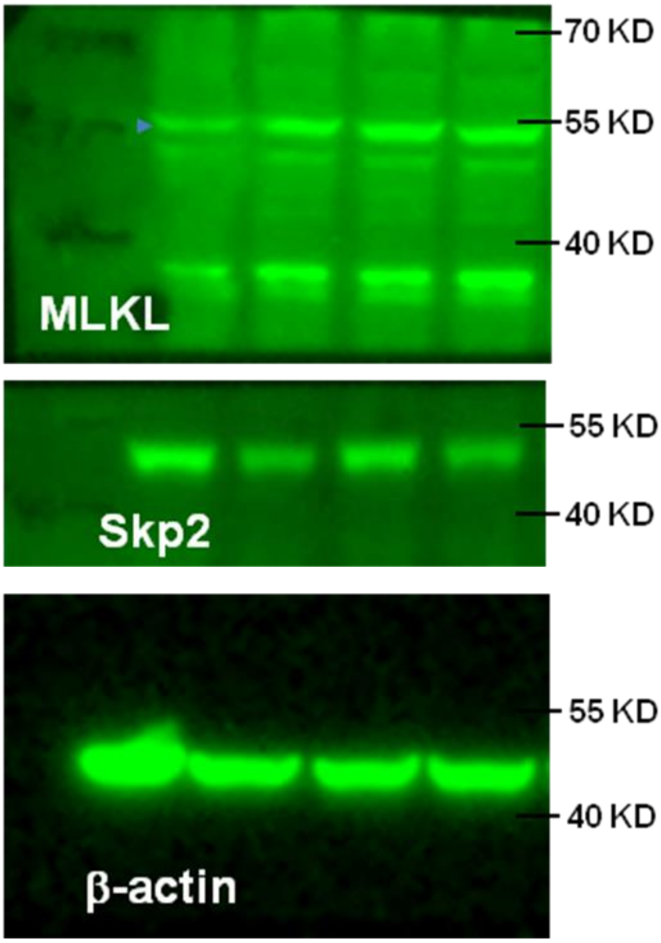

By FluorChem FC2 imaging system (Alpha innotech, USA)

Fig. 5b

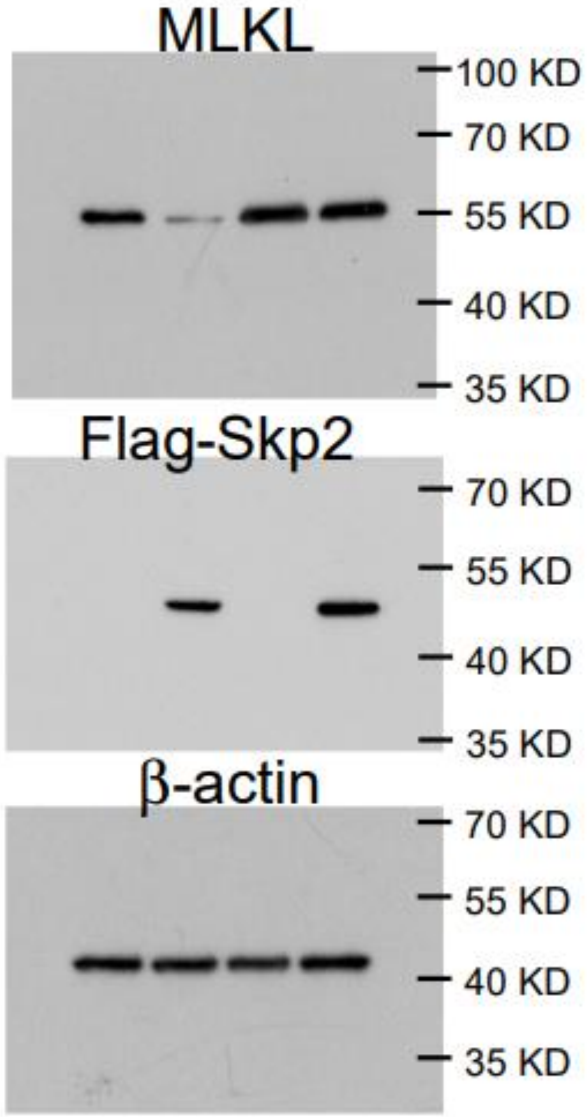
MLKL
100 KD
70 KD
55 KD
40 KD
35 KD
Flag-Skp2
70 KD
55 KD
40 KD
35 KD
β-actin
70 KD
55 KD
40 KD
35 KD

Fig. 5c

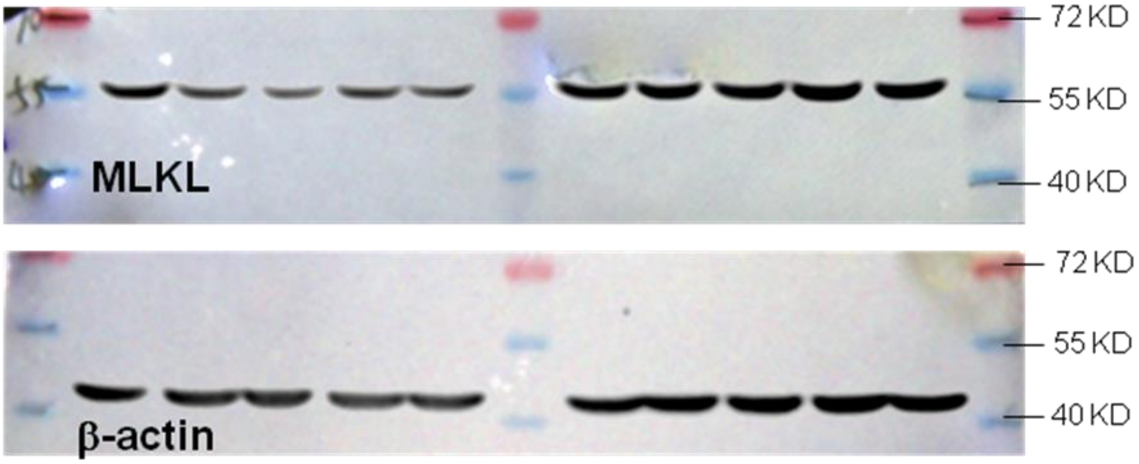

By AMERSHAM imageQuant800 system (GE, USA)

Fig. 5d

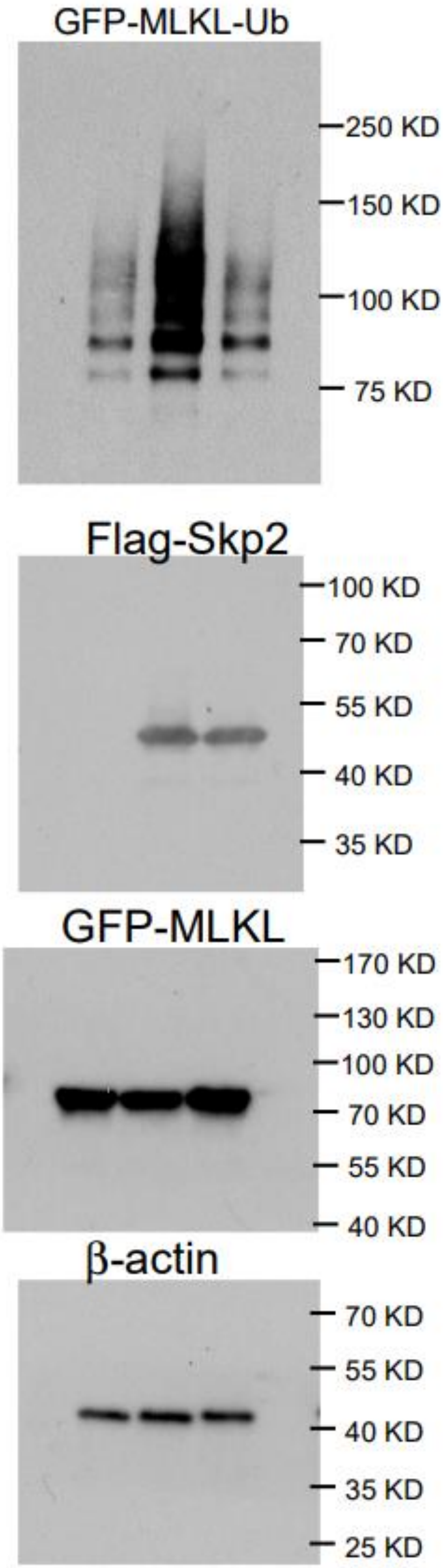
GFP-MLKL-Ub
250 KD
150 KD
100 KD
75 KD
Flag-Skp2
100 KD
70 KD
55 KD
40 KD
35 KD
GFP-MLKL
170 KD
130 KD
100 KD
70 KD
55 KD
40 KD
β-actin
70 KD
55 KD
40 KD
35 KD
25 KD

Fig. 5e

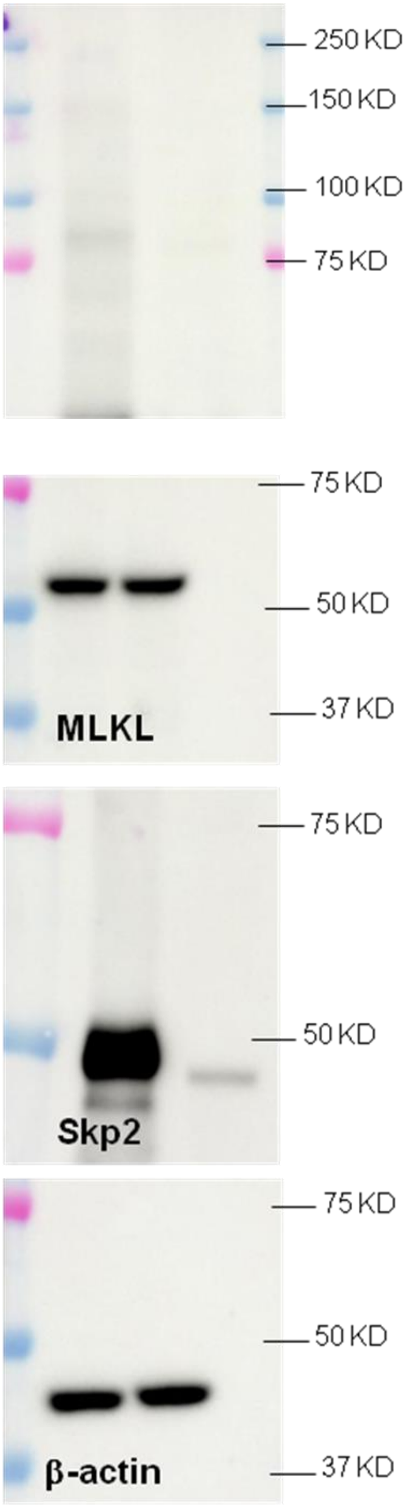

By AMERSHAM imageQuant800 system (GE, USA)

Fig. 6b

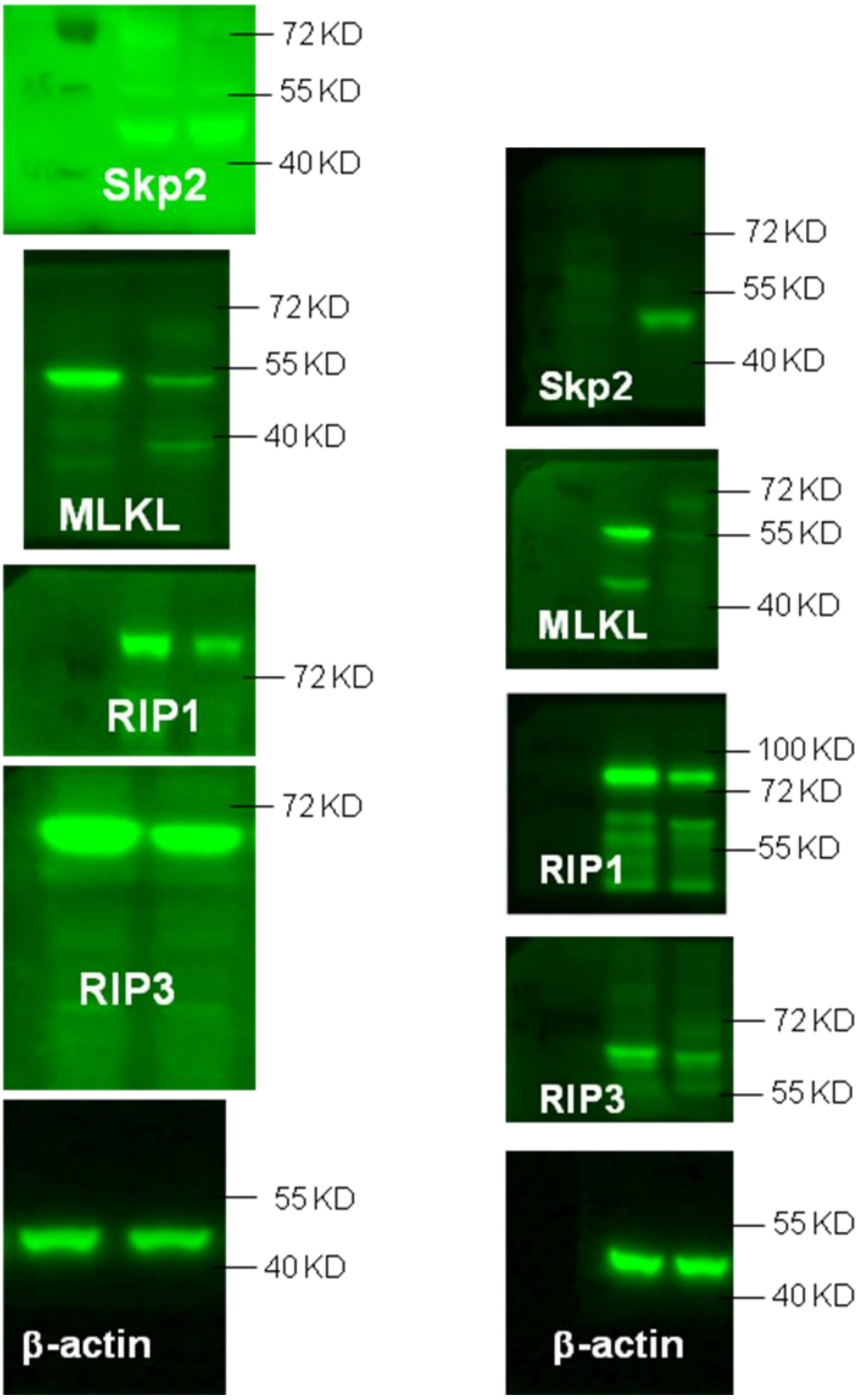

By FluorChem FC2 imaging system (Alpha innotech, USA)

Fig. 6c

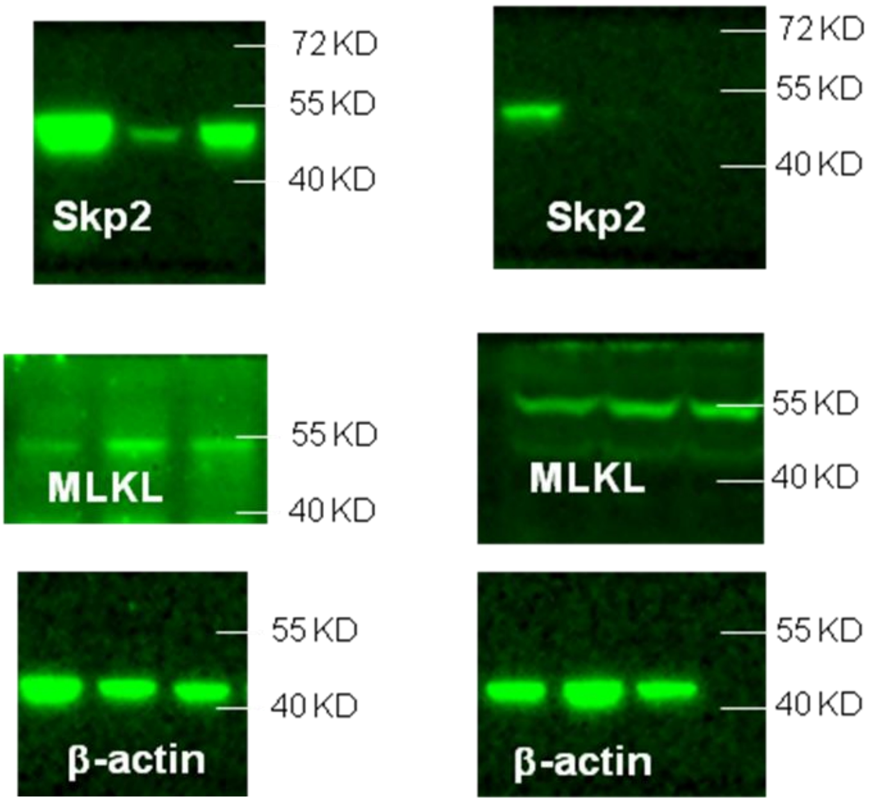

By FluorChem FC2 imaging system (Alpha innotech, USA)

Fig. 6f

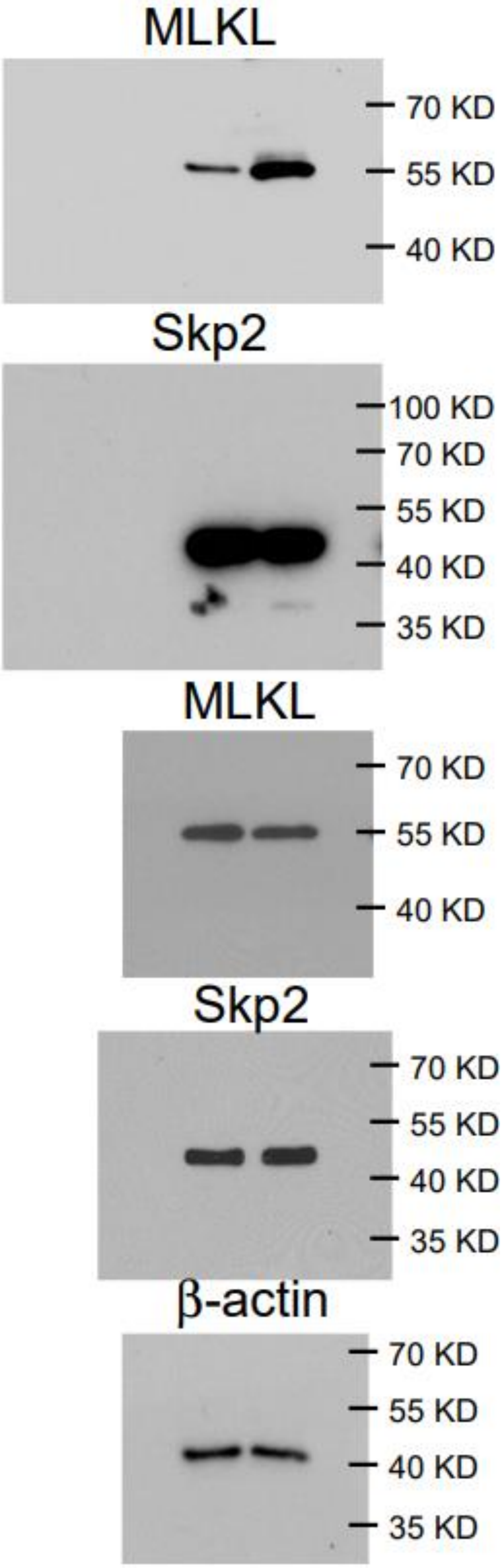
MLKL
70 KD
55 KD
40 KD
Skp2
100 KD
70 KD
55 KD
40 KD
35 KD
MLKL
70 KD
55 KD
40 KD
Skp2
70 KD
55 KD
40 KD
35 KD
β-actin
70 KD
55 KD
40 KD
35 KD

Fig. 6g

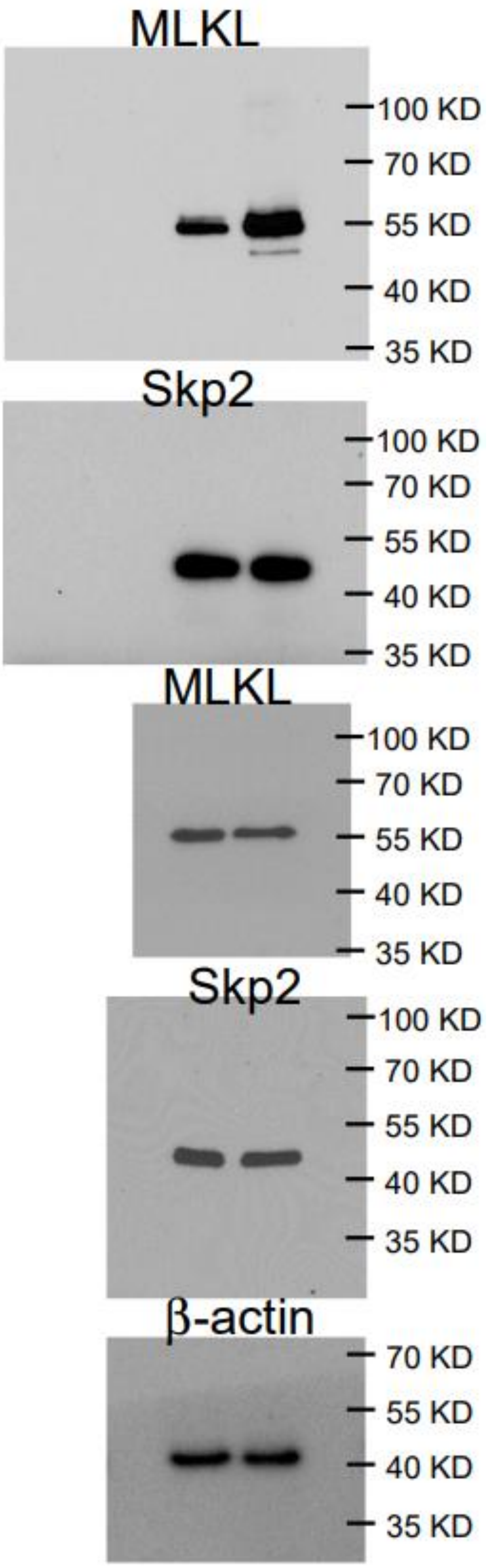
MLKL
100 KD
70 KD
55 KD
40 KD
35 KD
Skp2
100 KD
70 KD
55 KD
40 KD
35 KD
MLKL
100 KD
70 KD
55 KD
40 KD
35 KD
Skp2
100 KD
70 KD
55 KD
40 KD
35 KD
β-actin
70 KD
55 KD
40 KD
35 KD

Fig. 7a

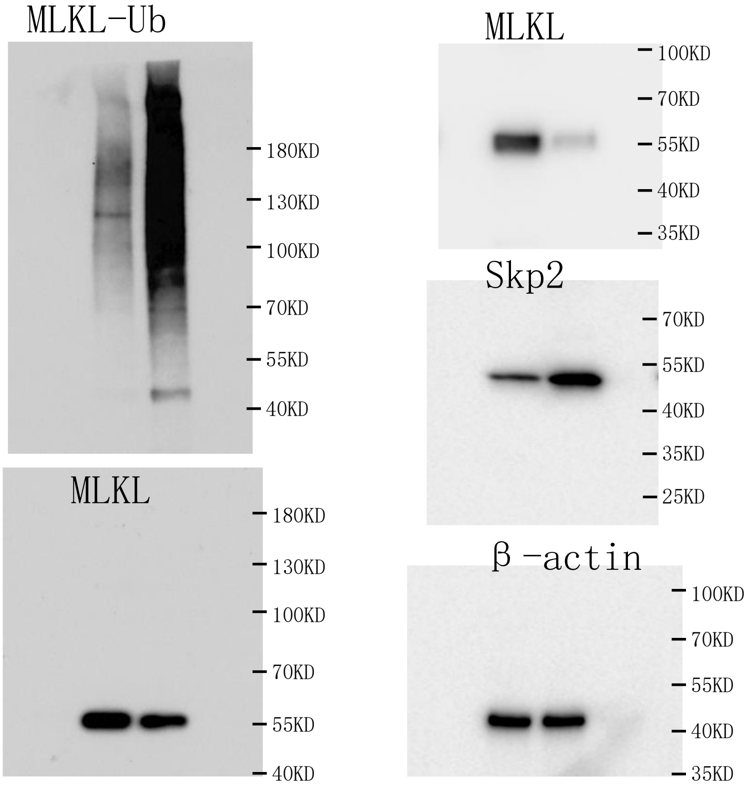
MLKL-Ub
180KD
130KD
100KD
70KD
55KD
40KD
MLKL
180KD
130KD
100KD
70KD
55KD
40KD
MLKL
100KD
70KD
55KD
40KD
35KD
Skp2
70KD
55KD
40KD
35KD
25KD
β-actin
100KD
70KD
55KD
40KD
35KD

Fig. S1a

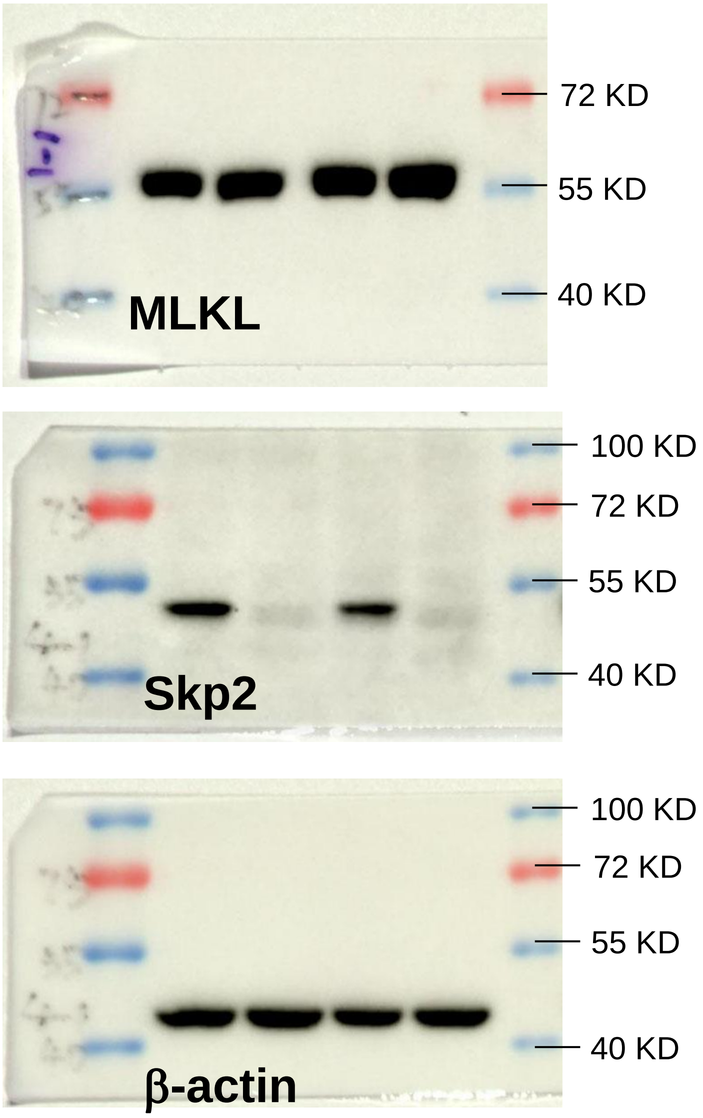

By AMERSHAM imageQuant800 system (GE, USA)

Fig. S1b

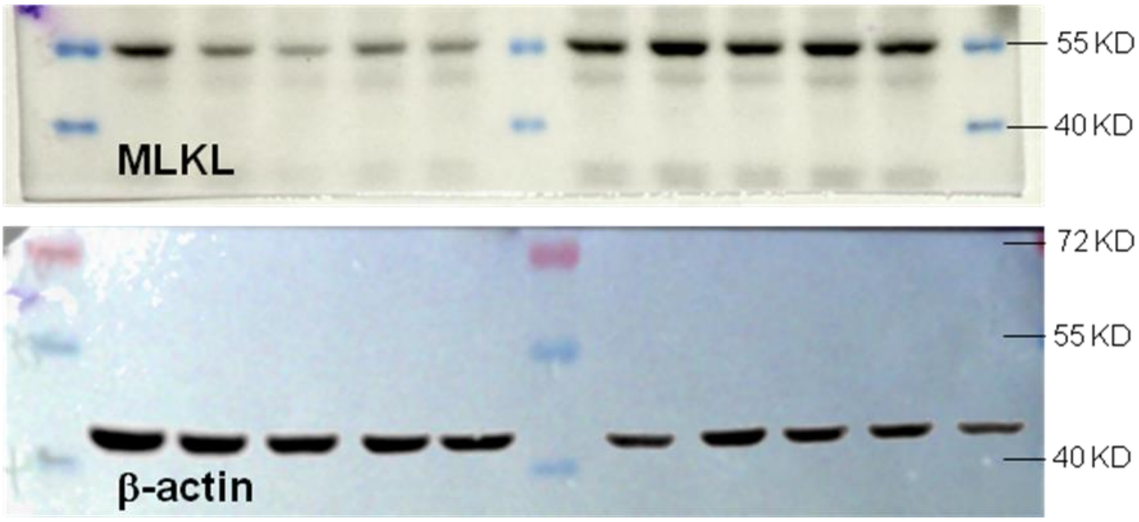

By AMERSHAM imageQuant800 system (GE, USA)

Fig. S1c

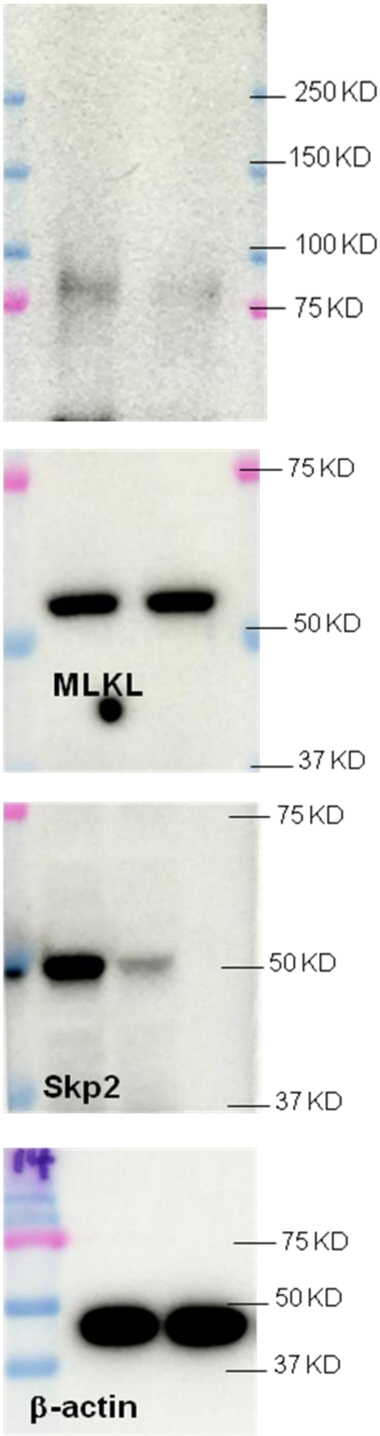

By AMERSHAM imageQuant800 system (GE, USA)

Fig. S2

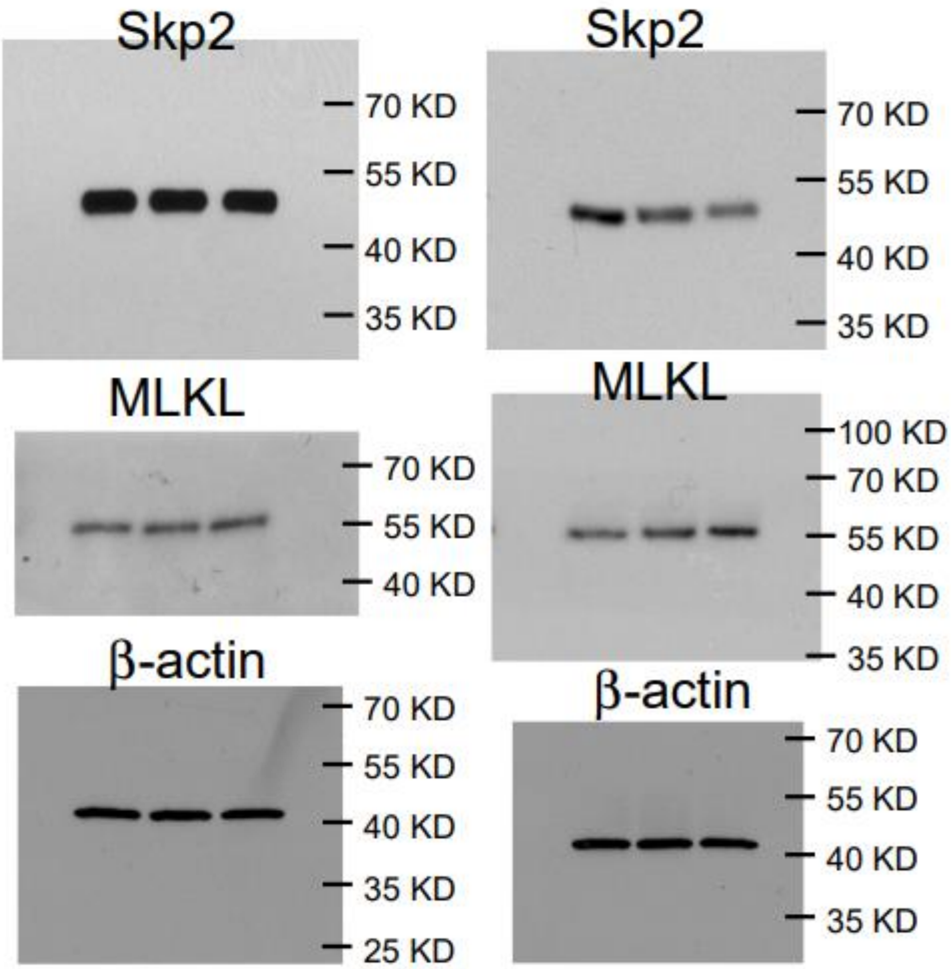
Skp2
70 KD
55 KD
40 KD
35 KD
Skp2
70 KD
55 KD
40 KD
35 KD
MLKL
70 KD
55 KD
40 KD
MLKL
100 KD
70 KD
55 KD
40 KD
35 KD
β-actin
70 KD
55 KD
40 KD
35 KD
25 KD
β-actin
70 KD
55 KD
40 KD
35 KD

Fig. S3

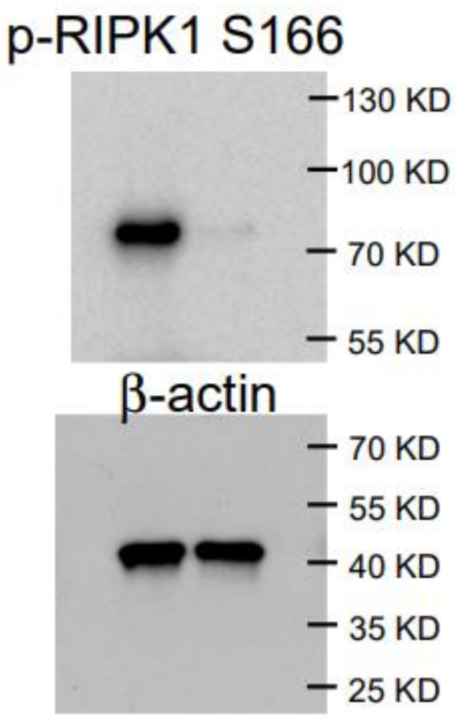

Supplementary Figure 4 uncropped and unedited blots and gels.
