# Supplementary material for: Skp2-mediated MLKL degradation confers cisplatin-resistant in non-small cell lung cancer cells
Source: Commun Biol. 2023 Aug 2;6:805. doi: 10.1038/s42003-023-05166-6 (PMC10397346; doi:10.1038/s42003-023-05166-6)
Supplement: Supplementary file 5 — Reporting Summary [file 42003_2023_5166_MOESM5_ESM.pdf]

nature portfolio

Corresponding author(s): Haidan Liu
Wei Li

Last updated by author(s): Jul 12, 2023

Reporting Summary

Nature Portfolio wishes to improve the reproducibility of the work that we publish. This form provides structure for consistency and transparency in reporting. For further information on Nature Portfolio policies, see our Editorial Policies and the Editorial Policy Checklist.

Statistics

For all statistical analyses, confirm that the following items are present in the figure legend, table legend, main text, or Methods section.

n/a	Confirmed	
☐	☒	The exact sample size (*n*) for each experimental group/condition, given as a discrete number and unit of measurement
☐	☒	A statement on whether measurements were taken from distinct samples or whether the same sample was measured repeatedly
☐	☒	The statistical test(s) used AND whether they are one- or two-sided<br>*Only common tests should be described solely by name; describe more complex techniques in the Methods section.*
☒	☐	A description of all covariates tested
☐	☒	A description of any assumptions or corrections, such as tests of normality and adjustment for multiple comparisons
☐	☒	A full description of the statistical parameters including central tendency (e.g. means) or other basic estimates (e.g. regression coefficient) AND variation (e.g. standard deviation) or associated estimates of uncertainty (e.g. confidence intervals)
☐	☒	For null hypothesis testing, the test statistic (e.g. *F*, *t*, *r*) with confidence intervals, effect sizes, degrees of freedom and *P* value noted<br>*Give P values as exact values whenever suitable.*
☒	☐	For Bayesian analysis, information on the choice of priors and Markov chain Monte Carlo settings
☒	☐	For hierarchical and complex designs, identification of the appropriate level for tests and full reporting of outcomes
☒	☐	Estimates of effect sizes (e.g. Cohen's *d*, Pearson's *r*), indicating how they were calculated

Our web collection on statistics for biologists contains articles on many of the points above.

Software and code

Policy information about availability of computer code

Data collection	The gene expression data and survival information were downloaded from the Gene Expression Omnibus (GEO) database, including GSE29013, GSE37745, GSE31210, and GSE157011 datasets.
Data analysis	Statistical analyses were performed using the statistical package SPSS (version 16.0 for Windows, SPSS Inc, Chicago, IL, USA) and GraphPad Prism (GraphPad 7.0, San Diego, CA, USA). Kaplan–Meier analysis was performed using "survival" (version 3.5-5) and "survminer" (version 0.4.9) packages based on R studio software.

For manuscripts utilizing custom algorithms or software that are central to the research but not yet described in published literature, software must be made available to editors and reviewers. We strongly encourage code deposition in a community repository (e.g. GitHub). See the Nature Portfolio guidelines for submitting code & software for further information.

Data

Policy information about availability of data

All manuscripts must include a data availability statement. This statement should provide the following information, where applicable:

- Accession codes, unique identifiers, or web links for publicly available datasets
- A description of any restrictions on data availability
- For clinical datasets or third party data, please ensure that the statement adheres to our policy

All data supporting the findings of this study are available from the corresponding author upon reasonable request. Uncropped and unedited blot/gel images are available in Supplementary Figure 4. Source data are available in Supplementary Data 1.

Research involving human participants, their data, or biological material

Policy information about studies with human participants or human data. See also policy information about sex, gender (identity/presentation), and sexual orientation and race, ethnicity and racism.

Reporting on sex and gender	N/A
Reporting on race, ethnicity, or other socially relevant groupings	N/A
Population characteristics	N/A
Recruitment	N/A
Ethics oversight	Approved

Note that full information on the approval of the study protocol must also be provided in the manuscript.

Field-specific reporting

Please select the one below that is the best fit for your research. If you are not sure, read the appropriate sections before making your selection.

☒ Life sciences ☐ Behavioural & social sciences ☐ Ecological, evolutionary & environmental sciences

For a reference copy of the document with all sections, see nature.com/documents/nr-reporting-summary-flat.pdf

Life sciences study design

All studies must disclose on these points even when the disclosure is negative.

Sample size	NSCLC tumor tissues and the corresponding adjacent non-tumor tissues for Western blot analysis were obtained from the Department of Thoracic Surgery at the Second Xiangya Hospital with written informed consent (n=22). NSCLC tumor tissues and the corresponding non-tumor adjacent tissues for immunohistochemical staining (IHC) analysis were obtained from the Department of Pathology at Xiangya Hospital and the Second Xiangya Hospital (n=39).
Data exclusions	N/A
Replication	N/A
Randomization	N/A
Blinding	N/A

Reporting for specific materials, systems and methods

We require information from authors about some types of materials, experimental systems and methods used in many studies. Here, indicate whether each material, system or method listed is relevant to your study. If you are not sure if a list item applies to your research, read the appropriate section before selecting a response.

Materials & experimental systems

n/a	Involved in the study	
☐	☒	Antibodies
☐	☒	Eukaryotic cell lines
☒	☐	Palaeontology and archaeology
☐	☒	Animals and other organisms
☒	☐	Clinical data
☒	☐	Dual use research of concern
☒	☐	Plants

Methods

n/a	Involved in the study	
☒	☐	ChIP-seq
☒	☐	Flow cytometry
☒	☐	MRI-based neuroimaging

Antibodies

Antibodies used

MLKL siRNA (sc-93430) was purchased from Santa Cruz (Dallas, TX). Antibodies against Skp2 (#2652, 1:1000), MLKL (#14993, 1:1000), RIP1 (#3493, 1:1000), phospho-RIPK1 Ser166 (#44590, 1:1000), RIP3 (#13526, 1:1000), Ubiquitin (#3936, 1:1000) and p27 (#3686, 1:1000) were obtained from Cell Signaling Technology (Beverly, MA). Antibodies against β-actin (A5316, 1:10000), Flag tag (F3165, 1:1000) and Flag-HRP (A8592, 1:10000) were from Sigma-Aldrich (St. Louis, MO). Antibody against GFP tag (TA150032, 1:1000) was purchased from OriGene (Rockville, MD). MLKL antibody (#GTX107538, 1:1000) was from GeneTex (Irvine, CA). MLKL antibody (#orb32399, 1:1000) was from Biorbyt (St. Louis, MO). RIP3 (NBP1-77299, 1:1000) was from Novus Biologicals (Littleton, CO). Phospho-MLKL Thr357 (#MAB9187, 1:1000) was from R&D Systems Inc. (Minneapolis, MN). Secondary antibodies, including anti-rabbit IgG HRP (#7074, 1:10000) and anti-mouse IgG HRP (#7076, 1:10000) were purchased from Cell Signaling Technology (Beverly, MA).

Validation

Validated.

Eukaryotic cell lines

Policy information about cell lines and Sex and Gender in Research

Cell line source(s)

Human NSCLC cells, including NCI-H23 (CRL-5800TM), NCI-H125 (CRL-5801TM), NCI-H460 (HTB-177TM), NCI-H520 (HTB-182TM), NCI-H1299 (CRL-5803TM), NCI-H358 (CRL-5807TM), NCI-H1650 (CRL-5883TM), HCC827 (CRL-2868TM) are from American Type Culture Collection (ATCC).

Authentication

The cell lines used were authenticated.

Mycoplasma contamination

Negtive.

Commonly misidentified lines (See ICLAC register)

N/A

Animals and other research organisms

Policy information about studies involving animals; ARRIVE guidelines recommended for reporting animal research, and Sex and Gender in Research

Laboratory animals

athymic nude mice

Wild animals

N/A

Reporting on sex

All mice used were female.

Field-collected samples

N/A

Ethics oversight

Approved.

Note that full information on the approval of the study protocol must also be provided in the manuscript.
